# Supporting and hindering environments for participation of adolescents diagnosed with autism spectrum disorder: A scoping review

**DOI:** 10.1371/journal.pone.0202071

**Published:** 2018-08-29

**Authors:** Beate Krieger, Barbara Piškur, Christina Schulze, Uta Jakobs, Anna Beurskens, Albine Moser

**Affiliations:** 1 Institute of Occupational Therapy, School of Health Professions, Zurich University of Applied Sciences, Winterthur, Switzerland; 2 Department of Family Medicine, CAPHRI School for Public Health and Primary Care, Maastricht University, Maastricht, The Netherlands; 3 Research Centre for Autonomy and Participation for People with Chronic Illness, Zuyd University of Applied Sciences, Heerlen, The Netherlands; University of Wollongong, AUSTRALIA

## Abstract

The influence of a person’s environment and its modifying potential on participation is well recognized for most childhood disabilities, but scarcely studied for adolescents with autism spectrum disorder (ASD). A scoping review was conducted, the aim of which was to map the existing literature about supporting and hindering environments for the participation of adolescents with ASD. Sources of scientific evidence were searched for in four databases. Inclusion criteria were the perspectives of adolescents between 12 and 21, families, peers, or significant others; ecologic validity; and a clear connection between environment and participation. The publication dates ranged from 2001 to 2014 and partly up to 2018. The International Classification of Functioning, Disability and Health (ICF) served as the guiding framework for inclusion/exclusion during the selection process. Thematic analysis was performed by five independent reviewers. Results were additionally validated by stakeholders. This scoping review identified 5528 articles, and finally included 31 studies. Two main themes were found: “providing security” indicates how the environment, and specifically the parental, physical, and informational environments, have a securing or intimidating effect. The second theme, “helping to connect”, indicates which environments support or hinder social relationships or social activities, and hence participation. An additional third main theme, “tension in participation”, relates to ambiguities that seem essential to understand participation or isolation of adolescents with ASD. Results show that participation is a value-laden concept. This research widens the field of dealing with adolescents with ASD, as it directs attention towards the responsibility of the environment regarding participation.

## Introduction

At least 0.65 to 1% of the world’s population is diagnosed with autism spectrum disorder (ASD) [1–3]. According to the Diagnostic and Statistical Manual of Mental Disorders, Fifth Edition Disorders (DSM-5) criteria, this causes “clinically significant impairment in social, occupational, and other fields of current functioning” (p.50)[4]. Based on this diagnostic manual, medical-functional research currently predominates over the bio-psycho-social focus [2]. With regard to the latter, few studies have covered younger children [5], or adults [6] with ASD, nor did they focus exclusively on adolescents with ASD [7].

Adolescence is a period of physical adjustments and role changes within the family and society [8]. The family becomes less important. Adolescents experience transitions, often in out-of-school contexts such as peer-relationships, increasing mobility, increased independence and moving into post-secondary education or work [9]. For the first time, individuals intentionally build and reflect on social and occupational roles offering guidance on expected behavior and responsibilities [8]. While some roles are inherent, such as those of son/daughter or sibling, others such as such as friend, hobbyist, or professional are actively chosen. Engagement in occupational roles frequently includes social interaction with others, which is affected in adolescents with ASD. It has long since been established that adolescents have to learn patterns of actions required for participation in society as adults [10]. Fifty percent of adults with ASD show poor participation outcomes with regard to work, friendship and independent living [11–14]. Research has shown that intensifying the participation of adolescents with ASD might lead to satisfactory participation in adulthood [15–17].

Adolescents with ASD participate 25% less in cooperative interactions during inclusive schooling [18]. They have a higher score for loneliness [19], are often bullied [20] and commonly experience social anxiety [21]. About 50% have no peer relationships outside of pre-arranged settings, and friendship level is reported to be low [22]. Few participate in social groups such as those engaging in recreational activities or attending religious services [22]. Attendance rates for postsecondary education [23], and vocational participation[24] and community participation are reported to be low [25].

The International Classification of Functioning, Disability, and Health (ICF) summarizes these situations under its bio-psycho-medical perspective as “participation”, defined as *“involvement in life situations”* (p 10) [26]. Participation indicates how an adolescent’s life is interwoven with the social life of their family, friends, community and society, and includes feelings of belonging and engagement as well as a societal perspective [27]. Participation comprises performing activity alone or in an social entity, the latter referred to by some authors as “social participation”[28,29]. However, as the ICF has been widely criticized for its lack of conceptual clarity [27,30–34] and does not, for example, distinguish between subjectively experienced and objectively observable participation [27,30–32,35] we here extended the ICF definition to cover adolescents with ASD by adding “*being engaged in and /or performing meaningful activities in occupational and social roles*.” We decided to use Hart’s [36] participation ladder for further theoretical support. It focuses on the participation of children and adolescents in terms of shared decision processes, and defines a graded ladder ranging from “manipulation” up to “shared decision making with adults”. Due to the weaknesses of their social interaction and communication, adolescents with ASD may be at special risk for being patronized, resulting in less shared decision-making.

According to ICF, participation is shaped and influenced by environments, defined as making up *“the physical*, *social*, *and attitudinal environment in which people live and conduct their lives*” (p 10) [26]. Environments can support or hinder participation in a dynamic interplay [30,37]. Introduced as a classification, ICF lacks an in-depth view on how this interplay is conceptualized [34,38]. Kaplan’s “reasonable person model” stresses the reciprocal aspects of person-environment interaction [39,40]. From the point of view of environmental psychology, it describes supporting environments as those addressing the human desires to explore, understand, enhance competence, be part of a solution, and participate with others towards meaningful goals. These desires are specially appropriate for the phase of adolescence. Hindering environments prevent persons, in this case adolescents with ASD, from participation.

Among children and young people with disability, environmental factors like social support or available services generally explain between 50% and 64% of the variations in participation frequency and in-depth involvement [41]. Research has identified the mediating role of the environment as it affects the participation of children with disability [41–43]. As regards participation in the community by children with disabilities, the most common facilitators found were social support by family and friends and geographical factors (location) [44] while the most common barriers included attitudes, physical environment, transportation, policies and lack of support from staff and service providers. Environment is also inherent in activity settings [45], which for adolescents include not only their home or school, but also public spaces, sports facilities or social media.

In the field of autism, however, this role of the environment has hardly been researched. A linking study aiming to develop an ICF core set for children and young people with autism extracted 1200 meaningful concepts from the literature [7]. Of these, only 41 were assigned to the domain of environmental factors, compared to 1131 in the activity and participation domains. Combined with the relative paucity of research in autism focusing on adolescence, this indicates a knowledge gap. Adolescents with ASD are exposed to a range of different and often unknown environments, due to necessary developmental transitions. Yet it is not known what environments support or hinder these transitions and participation in general. From the perspective of economics, any poor outcome for persons with ASD is socially counterproductive [46]. More information about the environment and participation is relevant for professionals who work with adolescents with ASD, as it can help them establish environments supporting participation or remove those hindering participation. Service managers can build on this knowledge to configure public services to the participation needs of adolescents with ASD.

The aim of this scoping review was therefore to map the existing literature about supporting and hindering environments for the participation of adolescents with ASD.

## Method

A scoping review methodology was applied [47,48] to map the available knowledge on a topic which covers multiple disciplines, and to identify key concepts, gaps in research, and sources of evidence in order to inform practice, policy making, and research [49]. Unlike a systematic review, a scoping review identifies the entire relevant scope of the literature, regardless of study design and methodological quality of the included studies [47,50] and is recommended when the research focus is broader. We used the five stages proposed by Arksey & O’Malley [47], and combined them with enhanced methodological rigor and the joint efforts of multiple reviewers, as recommended in different publications on the methodology for scoping reviews [48–51].

### Stage 1: Identify the research question

The following question was formulated: What is known in the scientific literature about the way environments support or hinder the participation of adolescents with ASD? We deliberately included early, middle and late adolescence, and so cover adolescents between 12 and 21 years of age, as there are differences in the legal age of adulthood in different countries.

### Stage 2: Identify relevant studies

The Scopus, Web of Science, Cinahl, and PsycINFO databases were systematically searched, concentrating on empirical studies, secondary reviews, or dissertations. Articles were limited to those published in English in peer-reviewed journals between 2001 and 2014, to include the publication date of ICF. Five main key terms, viz. adolescents, ASD, participation, environment, and influence, were comprehensively searched for. In the past two decades, there have been many changes to the way individuals with autism spectrum disorder are diagnosed. The present research is based on the 2013 DSM-5 [4] criteria. As previous editions used different criteria and subgroups, we also included DSM-4 [52] and ICD-10 [53] diagnostic terms to cover the whole historical spectrum of autism and regionally different diagnostic manuals. Additionally, terminology from the ICF classification [26] and the theoretical framework [39,40] generated broadening terms (Table 1). These were truncated, exploded, and adjusted to enable comprehensive coverage, as emphasized in scoping methodology [48]. The first author performed the search and documented the results by using Endnote® or Mendeley® as a data management tool. This facilitated merging double hits and reading the abstracts.

**Table 1:** Search strategy.

Main search terms	Additional broadening search terms^1^
adolescent*	youth, young adult*, student*, user*, pupil*, child*, teenager, adulthood
autism spectrum disorder*	autis*, ASD, Asperger syndrome, high function* autism (HFA), pervasive developmental disorder (PDD) “childhood disintegrated disorder”, Rett syndrome
**participation**	involve*, “life situation”, daily activit*, tasks, social competen*, communication, relationship, commun*, mobil*, “human need*”, engag*, “meaningful acti*,”human acti*", integration, inclusi*,
environment*	technology, “built environment”, “physical environment”, “social environment”, attitude, system, services, policies, context, “informational needs”, restoration, ecology, setting, ambient*, cultur*
influence*	impact, relat*, effect*,

*used with asterisk

^**1**^**based** based on DSM-4 and ICD-10 criteria, ICF terminology and the reasonable person model by Kaplan et al. [39,40].

An additional hand search in four autism-related journals (Autism Research, the Journal of Autism and Developmental Disorders, Autism, and Research in Autism Spectrum Disorders) was carried out for the years 2015–2018.

### Stage 3: Select studies

**Fig 1:**
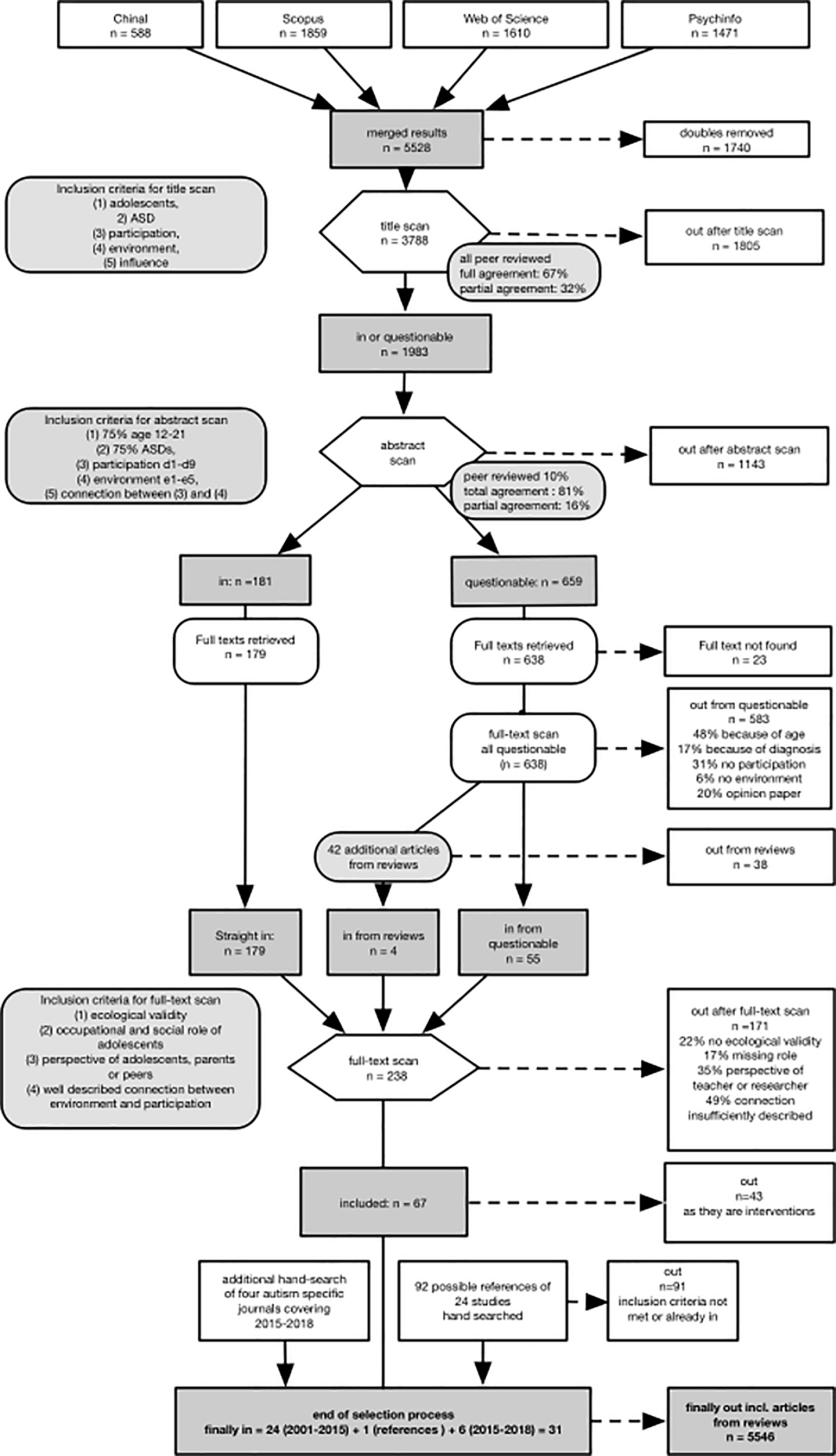
Flowchart of the selection process.

A peer-reviewed three-step selection procedure [47] based on title, abstract, and full-text levels was conducted, using inclusion/exclusion criteria. As the scoping review process is iterative, the researchers reflected on, defined, and re-defined the inclusion criteria post-hoc [51] after re-examining them. The final inclusion criteria for each of the three steps are presented in detail in the visual flowchart in Fig 1, as well as the reasons for exclusion. Each step graded the article as “in”, “questionable” or “out”. Studies graded as “in” or “questionable” were taken along to the next selection step.

After eliminating any doubles, the remaining titles were scanned (step 1) to see whether they addressed each of the five key aspects (adolescents, ASD, participation, environment, and influence). During the selection at abstract level (step 2), abstracts were included if (1) at least 75% of the study sample were adolescents between 12 and 21, (2) at least 75% had been diagnosed with ASD or all subordinate ICD-10 diagnoses, (3) the study focused on participation aspects included in ICF domains d1-d9, (4) the study described environmental factors included in ICF domains e1-e5, and (5) the article indicated any connections between these particular aspects of participation and environmental factors. Studies focusing on neurobiological, genetic, epigenetic, or pharmaceutical aspects were excluded, as well as those that were theoretical or descriptive in nature, or focused on functions (e.g. processing information) or personal factors (e.g. motivation). After retrieval of full texts, those graded in the abstract scan as “questionable” were re-scanned according to the abstract scanning criteria. In the case of reviews, all reviewed articles were scanned individually using the inclusion/exclusion criteria.

Finally, the first author read all remaining full-text articles, and applied the following agreed-on inclusion criteria for the full-text scan: (1) studies had to present ecological validity, i.e. the degree to which conditions used in the study are similar to those that would occur naturally [50]; (2) the aspect of occupational and/or social roles had to be included, and (3) studies had to include the perspectives of adolescents, their parents, or peers; and (4) there had to be a clear connection between participation and environment according to the definition established in advance. In some cases, the perspective of the adolescents could not be examined, (e.g. in nonverbal adolescents), and studies combining observations and parental perceptions were included to maintain a comprehensive scope. Studies purely involving observations by researchers or teachers were excluded. At the end of the selection process, reference lists of included studies were hand-scanned.

Procedural rigor [47,48] was secured by having the first author perform all the above steps. All co-authors (AM, BP, AB, CS, UJ) validated this process. The rates of agreement based on the title and the abstract scans are presented in Fig 1. When disagreement occurred, the titles were taken to the next step. Finally, the core team defined the inclusion criteria for the full-text scan, and decided together if connections between participation and environment in real-world settings were clearly described.

### Stage 4: Charting and analyzing the data

The data were charted using inductive thematic analysis [47,48]. According to Levrac and colleagues [48], *“thematic analysis requires additional description to assist authors in understanding and completing this step”* (p 8) and resembles qualitative data analysis techniques. Specifically, we extracted parts of sentences from the selected studies as described by Yoffe & Yardly [54]. These served as meaningful coding units if they expressed “participation”, “supporting environments”, or “hindering environments”. Eight studies were charted by all co-authors, resulting in a 50% agreement rate. Analysis of the differences, mostly in allocating coding units to “participation” or “environment”, helped to refine the definitions. Finally, 592 coding units (248 relating to participation; 194 to supportive environment; and 150 to hindering environment) were merged and mapped into themes and subthemes. All subthemes were defined separately and supported with quotes, which were subsequently reviewed and agreed on by the core team. Additionally, some of the analysis and development of themes was performed together with two co-authors (CS; UJ).

### Stage 5: Summarizing and reporting the results

Results are presented descriptively and narratively in the Results section below.

### Stage 6: Consultations: Validating with stakeholders

Consultations with relevant stakeholders are recommended to increase the rigor of scoping reviews [47,48]. Yet, descriptions of how to do this are generally vague. The authors presented the emerging results to three groups of stakeholders in connection with this research: adolescents and young adults with ASD, parents and professionals. Seven persons were asked whether the results reflected their experiences, if they disagreed or whether any aspects were missing. The findings are reported in the Results section.

## Results

### Summary of selected studies

The scoping selection process started with 5528 studies being retrieved from databases, 31 of which met the inclusion criteria. All 31 articles are listed in Table 2, indicating numbers of participants, research questions, methodology used, social roles, activities, and environment identified, and the main results. Column three provides information about combined intellectual disability as described in the studies.

**Table 2:** Matrix of included studies (4 pages).

Author^1^	N^2^	ID^3^	Aims	Design^4^	Participation		Environment	Main results
					Role	Meaningful activity	ICF domains	
**Andrews (2014)** **AU**	214 (f)	S	To describe the relationships between impairment and contextual factors and community participation for girls and women with Rett syndrome.	Quantitative research Parents and carers (n = 214) Questionnaires	leisure user community service user	Recreational, physical, skill-based social, or self-improvement activities	e1-e5 mobility, residential area, education of parents, community services, available time of parents	The frequency of participation in community activities by girls and women with Rett syndrome was influenced by factors including age, level of mobility, maternal education and degree of community support.
**Arnell** **(2018)** **SE**	24 (m: 17) (f:7)	N	To describe how adolescents with ASD perceive, experience and reflect on their participation in physical activities	Qualitative research Adolescents (n = 24) Interviews	leisure user	Physical activities, sports like cycling, team sport, running, swimming, gymnastics	e1, e3-e5 elements of nature, weather, social demands, relationships, attitudes	Besides intrinsic aspects of willingness, participants perceived external demands as essential for their participation. Perceived social demands were prominent, but predictability and natural conditions (weather, insects) also played a role.
**Baines** **(2012)** **USA**	2 (m)	N	To look at how youth with disabilities develop identities as learners through their experiences across social contexts.	Qualitative research Adolescents (n = 2) Interviews/observations	students peers sons	Interactions in debating clubs, classrooms, break.	e3-e4 Social relationships, attitudes, respect	Students’ stories unfolded and were influenced by disability labels across different contexts in ways that continued to shape their future life trajectories.
**Bentley (2008)** **USA**	1 (f)	S	To explore the lived experience of a child labeled as having severe disabilities, and her peers with and without disabilities, in an “inclusive” school environment.	Qualitative research Adolescent (n = 1), peers (n = 23), family, staff Observations/interviews	peer student friend	Communication, school activities, leisure activities, social relationship	e3-e5 social relationships, inclusive setting, attitudes, staff and services	Symbolic inclusion and inclusive pedagogical practices were found to be instinctively and effectively utilized by a child with Rett syndrome and her peers, though they were given exclusionary models by paraprofessionals, and limited opportunities for interaction.
**Brewster (2010)** **UK**	20 (m: 14) (f: 6)	N	To explore what children do in their leisure time, what they would like to do in future and what difficulties, if any, they encounter.	Qualitative research Adolescents (n = 20) Focus group interviews	leisure user	Computer games, surfing the internet, television, youth clubs, dance and foreign language classes, after-school club, snooker, paintball	e1-e4 transport, peer relationships, parents, neighborhood, attitudes	Needs and challenges to achieve an active and varied life outside of school and home environments are various. Predominating are a preference for solitary activities and no inclusion in local peer groups.
**Cox** **(2012)** **USA**	123 (m: 90) (f: 33)	N	To improve understanding of the difficulties individuals with ASD experience when learning to drive.	Quantitative research Parents (n = 123) Questionnaires	driver	Driving	e1, e3, e5 technical aids, teaching programs, parental relationship, services	Learning to drive presents a substantial challenge to individuals with ASD. Parents use a variety of strategies to support the learning process.
**Dixon** **(2013)** **AU**	2 (m)	N	To identify the perceptions of key stakeholders within two schools regarding the transition procedures before transition to high school.	Qualitative research Adolescents (n = 2), mothers (n = 2), teachers (n = 2) executives (n = 2) Interviews	student	Participation in school camps, orienting themselves, homework, getting new friends	e2-e5 visual aids, social relationships, attitudes, services	Both schools experienced difficulties with implementing successful ongoing strategies. These difficulties were linked to the lack of collaboration among all stakeholders, including the students. In fact, there was no student voice.
**Evans (2001)** **NZ**	1 (f)	S	To describe the experiences of a girl with Rett syndrome in an inclusive school and how social relationships create meaningful contexts for individuals with limited skills	Mixed methods research Adolescent (n = 1), peers, family, staff Observations/ interviews/focus interviews/survey	peer friend	Communication, sharing time, doing things together, asking about preferences	e3-e5 social relationships, attitudes, services and staff	The positive friendship experiences described did not occur spontaneously, nor were they due to social skills instruction. Instead, they were associated with observable social behavior by caregivers and peers who were extending their own repertoires to accommodate someone with a severe disability.
**Giarelli (2013a) USA**	13 (m: 10) (f: 3)	N	To describe the phenomenon of transition to community among adolescents and young adults with ASD.	Qualitative research Adolescents (n = 13), mothers (n = 13), teachers(n = 5), employers (n = 5) Interviews	transition to adulthood	Transitioning, seeking knowledge about one’s health problems, self-determination, work, job seeking, participation in the community.	e2-e5 physical environment, social relationships, attitudes, services	The core psychosocial problem of transition into the community is to stay afloat while feeling “adrift". Adolescents, with the support of parents, teachers and sympathetic employers, used structuring, anchoring, and embarking to solve their problem.
**Giarelli (2013b) USA**	14 (m: 10) (f: 4)	N	To explore the perspectives of adolescents with regard to their own expectations and ideas on ways to facilitate successful transitioning.	Qualitative Research Adolescents (n = 14) Telephone interviews	transition to adulthood	Transitioning tasks and preferences	e3-e5 physical environment, social relationships, attitudes of staff, availability of services	Perceived barriers were: self-assessed behavioral problems, self-assessed associated features, other personal factors, and institutional factors. Bridges to facilitate transition were: facilities in the community, cognitive abilities, personal qualities/ strengths, and mentor's qualities.
**Gregor** **(2018)** **CA**	10 (m: 9) (f: 1)	N/A	To explore how social, personal, systemic, attitudinal and familial mechanisms influence physical activity participation among Canadian adolescents with ASD	Qualitative research Parents (n = 10) Interviews	leisure user	Physical activities, sports, alone or in groups	e1, e3—e5 accessibility, parental priorities, funding, attitudes, services,	Parents prioritized interventions over physical activities, which shaped interest in and experiences with physical activities. Further challenges were access to programs, lack of awareness of ASD among service providers, funding and limited program options
**Howe** **(2016)** **UK**	16 (m: 12) (F: 4)	N/A	To explore how adolescents with ASD perceive sensory differences to affect their learning experiences within the classroom	Mixed-methods research Adolescents (n = 16) Questionnaires Interviews	student	Learning at school, listening, concentrating	e2 physical environment	Participants reported difficulties in at least one sensory domain, with hearing affecting them the most. Content analysis revealed that sensory sensitivity affected the participant’s learning and that sensory experiences were largely negative.
**Humphrey** **(2008)** **UK**	20 (gender un-known)	N	To explore the views and experiences of pupils with AS about mainstream education and to identify practices that facilitate or constrain learning and participation	Qualitative Research Adolescents (n = 20) Interviews, diaries, drawings	pupil peer	Activities in school like communicating, interacting with peers, learning, transport to school (taxi)	e3-e4 peer relationships, social relationships attitudes	The central theme was how participants constructed their understanding of what their AS meant to them. The links between this understanding and reported difficulties with peers and teachers are described. The desire to ‘fit in’.
**Humphrey** **(2010)** **UK**	40 (gender un-known)	N	To examine the frequency of bullying and levels of social support in pupils with ASD and two control groups. To examine the contribution of social support to the frequency of bullying.	Quantitative research Adolescents (n = 40) Peers (n = 80) Questionnaires	peer student	Being kicked, threatened to be hurt, being demanded to hand over money, others trying to hurt them, to break sth. or to hit them.	e3-e5 peer relationships, social relationships, attitudes, services, policies	Pupils with ASD experienced higher frequency of bullying and lower levels of social support from most interaction partners. Receiving support from classmates was the most important means of reducing the frequency of bullying.
**Kuo** **(2011)** **USA**	91 (m: 74) (f: 18)	M	To investigate friendship characteristics, agreement between adolescent’s and parent’s perceptions of the adolescents’ friendships and factors that may be associated.	Quantitative research Adolescents (n = 91) Parents (n = 91) Questionnaires	peer friend	Eating, doing physical activities, outdoor activities, education-related activities, conversations, surfing websites, and many other activities	e3 Social relationships, friends,	Adolescents with an ASD and their parents identified different peers as the adolescent’s friends. The findings also reveal similarities and differences in friendships between adolescents with an ASD and typically developing adolescents.
**Kuo** **(2014)** **USA**	91 (m: 74) (f: 18)	M	To describe how adolescents with ASD use media, with whom they spend time using media, and the association between media use and parent–child relationships and friendships.	Quantitative research Adolescents (n = 91) Parents (n = 91) Questionnaires	media user peer friend	Different activities in connection with media use.	e3 social relationships peers, social networks, family	They most frequently watched cartoons, played computer or video games that involved shooting, and visited websites about video games. Those who watched television with parents reported more positive parent–child relationships. Those who visited social networking websites or received emails from friends reported more positive friendships.
**Liptak (2011)** **USA**	725 (m: 595) (f: 131)	M	To describe social participation and to identify factors that affect it.	Quantitative research Parents (n = 725) Questionnaires	peer student employee driver	Contact through phone calls, instant messaging, meeting friends outside organized gatherings, attending work or secondary school	e3, e5 social relationships, parents, financial resources	Although many graduated from high school and integrated into society, many became increasingly isolated and had poor rates of employment and postsecondary education. Positive effects on outcomes included the ability to communicate effectively, coming from an environment that is not impoverished, and having parents who advocate.
**Locke (2010) USA**	7 (m: 4) (f: 3)	N	To examine the social–emotional relationships of adolescents with autism and their typically developing classmates in a mainstream high school.	Quantitative research Adolescents (n = 7) Peers (n = 13) Questionnaires	friend peer student	Five domains of friendship quality, including companionship (spending time together) help, security, conflict and closeness	e3-e4 peer relationships, attitudes,	Adolescents with autism experienced significantly more loneliness than their typically developing classmates, had significantly poorer friendship quality regarding companionship and helpfulness, and had significantly lower social network status than their typically developing classmates.
**Lounds Taylor** **(2017)** **USA**	36 (m. 30) (f: 6)	M	To examined how social participation changed after youth with ASD exited high school. To explode the interrelations between types of activities (structured vs. unstructured activities).	Quantitative research Parents (n = 36) Questionnaires	peer friend neighbor religious service user social actor in public space leisure/sports	Unstructured activities like meeting relatives, peers or friend Structured activities like attending religious services and sport	e5 attending school	Results confirm that youth with ASD might be at-risk after leaving high school–by becoming more isolated from structured social activities. Many youth maintained their levels of contact with friends, neighbors, and extended family members, youth with more internalizing symptoms appear to be at greatest risk for experiencing declines in both structured and unstructured activities.
**Muskat** **(2016)** **CA**	20 (m: 17) (f: 3)	M	To understand the hospitalization experiences of children and youths with ASD, their families, and their health-care providers (HCPs) with the objective of utilizing the findings to improve hospital care for children and youths with ASD.	Qualitative research Adolescents (n = 6) Parents (n = 22) Heal care professionals (n = 14)	patient in a hospital	Waiting, talking with medical staff, undergoing examinations	e2-e5 relationship with medical staff, physical environment, attitudes, services	Problems in the context of health-care delivery in the hospital setting included communication and sensory challenges, and the degree of flexibility of HCPs and the hospital organization. Supportive HCPs acknowledged parents as experts, inquired about the requirements of patients with ASD and implemented strategies that accommodated the unique clinical presentation of the individual patient.
**Myers** **(2015)** **USA**	17’818 (m: 83%) (f: 17%)	S: 30% M: 27% N: 41%	To examine the influence of extrinsic influences such as socioeconomic status, school location, transportation, and case management, as well as intrinsic characteristics, on social and community participation by adolescents with ASD transitioning into adulthood	Quantitative research Parents (n = N/A) Adolescents (n = N/A) Teachers (N = N/A) School administrators (n = N/A) Questionnaires	peer leisure user public peer transitions to adulthood	Community participation, social participations	e3-e5 socioeconomic status, school location, transportation, services	Community participation was associated with factors related to family resources, household income and utilization of case management. Social participation outcomes appeared to be more associated with factors that are inherent to the individual. Individuals with ASDs rely on others to help organize community and social opportunities, putting them at higher risk of poor outcomes if there is no such advocate across the lifespan.
**Orsmond (2004) USA**	185 (m: 140) (f: 45)	M	To describe friendships and peer relationships in social and recreational activities. To examine predictive individual and environmental factors for this.	Quantitative Research Mothers (n = 185) Questionnaires	friend leisure user	Friendship (defined with ADI-R) leisure activities	e3-e5 peer relationships, parents, attitudes, services and inclusive schooling	No environmental factor was a significant predictor of having peer relationships. Participation in social /recreational activities was sensitive to environmental factors like services received, maternal participation in social/recreational activities, and inclusive schooling.
**Orsmond (2006) USA**	202 (m: 147) (f: 54)	M	To examine mother–child relationship in families of adolescents and adults with an autism spectrum disorder	Mixed methods research Mothers (n = 202) Questionnaires, interviews	son daughter	Verbally expressed warmth and positive affect and gains and strains	e3 mother-child relationship	Over 90% of the mothers reported to have a warm relationship characterized by high levels of affection for their child. Alteration of social impairment does not fundamentally impair the mother–child relationship during adolescence and adulthood.
**Petalas (2013)** **UK**	12 (m:11) (f: 1)	N	To explore the perceptions and experiences of adolescents with ASD growing up with a brother or sister without ASD	Qualitative research Adolescents (n = 12) Interviews	sister brother	Interactions like shared activities, enjoyment, conflicts and identity construction	e3 social relationship to siblings	Adolescent siblings with an ASD experience a world that is much like that of typically developing siblings, but where differences may be obvious they are often reminded of their “differentness”.
**Pisula (2012)** **PO**	25 (m: 22) (f: 3)	N	To determine how adolescents with AS perceive their social situation with peers at school, focusing on attitudes.	Quantitative Research Adolescents (n = 25) Peers (n = 25) Questionnaires	peer	Support, sense of security, being appreciated, readiness to engage, providing help, lending money, giving feedback, aggressiveness, sociability	e3, e4 peer relationship, attitudes	Adolescents with AS rated their classmates’ attitude towards them and their attitude to their classmates lower than typically developing adolescents. They also claimed to receive less support from classmates. The type of support correlated with their peers, attitudes towards them.
**Rossetti (2011)** **USA**	2 (m: 1) (f: 1)	M	To explore the contexts and dynamics of friendships between two individuals with autism and peers without disabilities.	Qualitative research Adolescents (n = 2) Peers (n = 3) Interviews	friend	Communication patterns, school activities like academic work, dancing classes, breaks and leisure activities, conversations	e3 peer relationship	Students without disabilities invest more efforts to sustain connections as friends and take opportunities to interact. The findings show how students with and without disabilities enacted meaningful relationships
**Ryan** **(2010)** **UK**	48 (gender un-known)	M	To focus on the emotional work parents do during participation in public places with their children with ASD	Qualitative research Parents (n = 48) Interviews	social actor in public space	Eating in restaurants, buying goods in a store, attending cinema, managing public encounters	e2-e4 parental relationship, social relationships, attitudes, physical environment	It is a complex emotional labor when parents go out in public with their children with ASD. They try to manage the unpredictability, their children’s distress and the responses of others. Each setting is socially, spatially and temporally unique.
**Saggers (2011)** **AU**	9 (m: 7) (f: 2)	M	To examined inclusive education practice from the perspective of the student and identify practices that facilitate and constrain learning and participation.	Qualitative research Adolescents (n = 9) Interviews	student pupil peer	Academic activities, enacting friendships, relationships to teachers, supporters and peers, homework, handwriting, technology use, workload	e1-e5 teaching materials, space and architecture, social relationships, attitudes, services	Six characteristics of successful inclusive schools emerged: committed leadership, democratic classes, reflective educator, supportive school culture, engaging and relevant curricula, responsive instruction.
**Shattuck (2011)** **USA**	900 (m: 760) (f: 140)	M	To explore the rates of participation in social activities among adolescents with an ASD compared to adolescents with other disabilities.	Quantitative research Parents (n = 920) School staff (n = unknown) Questionnaires	friend volunteer sportsperson leisure user	Seeing or calling friends, invited activities, performing volunteer services, taking lessons or classes, non-school activities, groups they belong to	e3-e5 social relationships, peer relationships, attitudes, finances,	Adolescents with an ASD were significantly more likely to never see friends out of school, or to get called and be invited to social activities. Correlates of limited social participation included low family income, impairments of conversational ability, social communication, functional cognitive skills.
**Symes (2010)** **UK**	120 (m: 29) (f: 11)	N	To investigate sociometric status, perceived levels of peer support and frequency of bullying experienced by pupils with ASD in secondary schools, compared to others	Quantitative research Adolescents (n = 40) Peers (n = 80) Questionnaires	peer pupil	Working and playing with partners. Experiences of bullying, experiences of support by friends	e3, e4 peer relationships, attitudes	Pupils with ASD were more likely to be rejected and less likely to be accepted by their peers, experienced higher levels of bullying, and reported lower levels of social support from classmates and friends than matched pupils with dyslexia or no special needs.
**Wainescot** **(2008)** **UK**	57 (m: 55) (f: 2)	N	To explore how and where pupils with AS/HFA spend their school day socially, both in and out of lessons.	Quantitative research Adolescents (n = 57) Questionnaires Pedometer	student peer	Having lunch, spending breaks, socializing with peers	e2, e3, social relationships, space and architecture	Pupils with AS/HFA engaged in fewer social interactions, both in and out of lessons, spent break and lunch times inside in quieter and supervised areas, had fewer friends, were less physically active and were more often the targets of bullying than matched peers

^1^ First author, date, country

^2^ Number of participants and gender

^3^ Co-occurring intellectual disability (ID) : N = no ID reported M = mixed ID reported; S = strong ID reported

^4^ Design, participants, data collection

Most of the studies were found in two databases simultaneously, and the majority (76%) of the articles were published after 2010. The types of journals, the countries where the studies were performed, and information about the designs is provided in Table 3.

**Table 3:** Patterns of publications 2001–2018.

Main aspect	Distribution	n = 31	%
**Database**	Scopus (2001–2014)	9	29
	Web of Science (2001–2014)	5	16
	Both Scopus and Web of Science (2001–2014)	6	19
	Scopus or Web of Science with either		
	PsycINFO or Cinahl (2001–2014)	6	19
	Hand search in reference lists	1	1
	Hand search in journals of autism (2015–2018)	6	19
	PsycINFO or Cinahl (2001–2014)		19
**Publication date**	2001–2009	6	19
	2010–2014	19	61
	2015–2018	6	19
**Types of journals**	Disability studies	5	16
	Medical (incl. those specializing in autism)	14	45
	Educational science	9	29
	Others	3	10
**Countries**	United States and Canada (2 mixed)	17	54
	United Kingdom	8	26
	Australia	3	10
	Poland	1	1
	New Zealand	1	1
	Sweden	1	1
**Study design**	Cross-sectional or mixed design	16	52
	Qualitative design	15	48
**Time-range of collected data**	Current data	26	84
	Retrospective data	5	16

Table 4 offers more detailed information about the content of the 31 studies. Results comprise a total of 20,768 adolescents with ASD. The number of participants ranged from 1 [55,56] to 17,818 [57]. Of these, 81% were male and 19% female, while some did not specify gender. Low (27%), mixed (35%) and no intellectual disability (36%) were nearly evenly distributed. Of the known data ([57] missing), 79% was provided by parents, whereas adolescents with ASD provided 18%, and peers 7%. The majority described school settings (59%), whereas community settings (35%) and home settings (21%) were less often described. Environments as defined in the ICF classification included products and technology (11%), natural or human-made changes to the environment (10%), support and relationship (38%), attitudes (20%), and services, systems, and policy (19%). In all, 13 different social roles of adolescents with ASD were identified. The form of participation described covered interpersonal relationships (31%), major life areas (37%), and community, social and civic life (13%), while all other ICF participation domains covered less than 2% each.

**Table 4:** Characteristics of adolescents, environments and participation in 31 studies.

**Main aspect**	**Distribution**	**N (participants)**	**%**
**Adolescents with ASD**	Total number of participants	20,768	100
	Male	16,906	81
	Female	3741	19
	Gender unknown	121	0.5
**Reported intellectual disability**	Strong intellectual disability reported	5667	27
	Mixed intellectual disability reported	7467	35
	No intellectual disability reported	7647	36
**Informants (n = 3392)**	Adolescents	402	18
	Parents	2703	79
	Peers	249	7
	Others	38	1
**Main aspect**	**Distribution**	**N (studies: double possible) %**	
**Settings (n = 37)**	School setting	16	59
	Home setting	8	21
	Community settings (e.g. leisure, public spaces hospital, church, work)	13	35
**ICF environmental domains (n = 62)**	e1 (products and technology)	7	11
	e2 (natural and human-made changes)	6	10
	e3 (support and relationships)	24	38
	e4 (attitudes)	13	20
	e5 (services, systems, and policy)	12	19
**Social and occupational roles (n = 56)**	Student/pupil	14	25
	Peer	11	20
	Friend	10	19
	Leisure participant (incl. sport)	6	10
	Public space user	4	7
	Son/daughter	2	4
	Brother/sister	1	2
	Driver	1	2
	Media user	1	2
	Employee	1	2
	Social roles in transition to work	3	5
	Religious service user	1	2
	Patient in a hospital	1	2
**ICF participation domains** **(n = 54)**	d7 (interpersonal interactions and relationships)	17	31
	d8 (major life areas)	20	37
	d9 (community, social and civic life)	7	13
	d1-d6 less than 5% each	1–2 each	<2

### Supporting and hindering environments for participation

**Fig 2:**
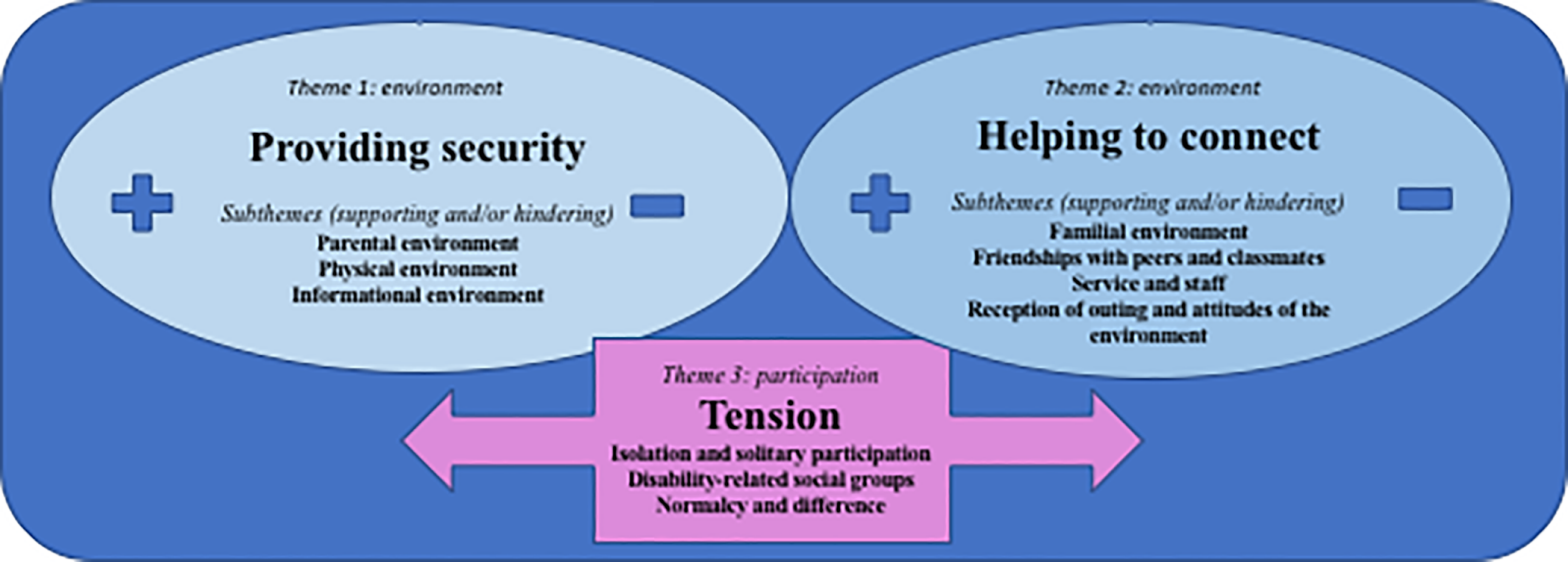
Graphic representation of supporting and hindering environments and tension in participation.

Thematic analysis of supporting and hindering environments for participation revealed two main themes, “providing security” and “helping to connect”. They are described here with seven subthemes (see Fig 2), each of which comprises supporting and hindering aspects.

The first main theme, **“providing security”,** describes how their environment provides adolescents with personally perceived security to support their participation, or hinders it by neglecting security issues, resulting in intimidation and even fear. Three subthemes were found crucial in providing security.

The first subtheme, “parental environment”, refers to the way parents provide security and protection as a precondition for participation. The stability of a supportive relationship with parents has been described as “anchoring” when transferring to new participation areas like work, college, or new places [58–60]. More specifically, higher self-esteem and optimism on the part of the mother contributes to participation [58,59], as it encourages taking risks and developing positive connotations about participation. Hence, the stability of the relationship with the mother during adolescence and adulthood is described as crucially important [58]. A further supportive aspect is when parents understand and adapt to the security needs of their children [58,60–63]. Adolescents with ASD clearly expressed their preference for “safe” activities. For instance, one boy stated that he would only leave the house for communal leisure participation after his parents had checked that it was safe [62]. As regards the location of activities, adolescents with ASD prefer home-based activities like watching television, playing in the garden, playing computer games, or surfing the Internet. This tendency becomes even stronger as they grow older [57,62]. To provide security and thus support participation, it is prudent for parents to explore environments beforehand [64–66]. For example, they may check out a future school environment, or they can inform themselves about assisting programs for learning to drive. When parent fail to obtain this kind of information as a security function, they hinder their children’s participation [60,65].

The second subtheme, “the physical environment”, refers to the impact that physical aspects of the environment, such as noise, light, smells, space, and crowdedness have on the security perception and participation of adolescents with ASD. Physical environments involving bright lights, unusual noises, darkness, crowds, queues, or unfamiliar places are often described as intimidating and overwhelming, thus as hindering participation [60,61,66–68]. Authors of two studies judged noise sensitivity to be the chief problem [66,69]. Pupils at school lose concentration, which affects learning [69]. In public spaces, such as during transportation, or when attending a cinema, adolescents feel offended, disrupted, and thus victimized, when they are passively exposed to the overwhelming condition of noise. In school settings, calmer spaces like libraries are reported to be preferred by adolescents with ASD [70]. As regards outdoor physical activities, certain elements of nature, such as insects or weather conditions, are reported as barriers [71], while in hospitals, being touched frequently and hearing unusual noises are perceived as discomforting or can evoke panic [72]. Control of noise, but also of lighting or spaces provides security through agency. Availability of quiet areas such as school libraries, light dimmers, volume control of sound systems in public spaces, and repositioning of chairs to control space are supportive environmental conditions [59,66–68].

The third subtheme, “informational environment”, addresses the comprehensibility of environments. Adolescents with ASD often report that awareness of upcoming activities, social expectations, and implicit demands are challenging [64,65,71,73,74] Order and predictability are described as a *“security blanket” (p*.*35)* [68], as they mean that demands are more familiar and thus clearer. A higher participation rate has been reported for regularly scheduled and structured activities [22,75]. New situations, for example when starting a new job or attending a new sports facility, are described as a source of unease and instability [66,68,71,73,76]. Accessible and processable information become imperative environmental conditions for participation. Knowing what to expect beforehand allows these adolescents to plan their participation and engagement in physical activities[71]. When understanding, interpreting, and reacting to social demands are difficult for these adolescents, they feel intimidated by their environment [66–68,77]. It is difficult, for example, to understand the subtleties of social interaction between peers, or to perform multitasking demands (e.g. merging into traffic and trying to maintain speed while driving (92%)[65], listening and writing simultaneously [67]), or working according to tight schedules) [67]. Adolescents with ASD used words like “*drowning”* for this overwhelming experience (*p*.*229)* [59]. Providing information sequentially, for example as visual cues without time limits, allowing typing instead of handwriting, or teaching skills in small steps, ensures that information can be processed and thus provide both security and participation [18,64,65,67]. For the same reason, preparing adolescents mentally for new participation challenges supports participation. Additionally, adolescents with ASD know little about ways to access leisure activities [62]. After they leave high school, structured social activities of adolescents with ASD decline significantly [75]. Informing them beforehand, for example using video games or toy tractors as a basis for learning to drive, or visiting a new school before the transition is made, have also been described as supportive [64–67]. Since bullying is a frequent participation issue for adolescents with ASD, information about anti-bullying policies needs to be provided explicitly to these adolescents. This includes explanations of effective implementations, as an understanding of both contributes to their sense of security and supports participation [67].

The second main theme, “**helping to connect”**, addresses how environments help to create and strengthen social relationships with others, and contribute to friendship and a sense of belonging among adolescents with ASD. Four different subthemes have been found to be central in helping them to connect.

The first subtheme, “familial environments”, refers to conditions provided by family, parents, siblings, grandparents, wider family members, or family friends. Besides siblings, these are mostly adult social partners who support the aim of social participation and activities in the community. Shared family activities like watching TV, attending church services, or performing leisure activities, have been described as supportive for participation [22,73,76,78,79]. Specifically, community participation like going shopping or engaging in sports seems directly linked to a sharing familial environment [59,60,73,76]. This might be the reason, since in a large sample, the community participation rate did not drop after leaving school [57]. The same effect has been reported for unstructured social participation after leaving high school [75]. Others have reported how the company of a familiar person supports attending physical activities [71]. Additionally, a higher participation rate in leisure activities was found when the family climate was more sociable [22]. An important environmental condition for performing activities together is interests shared by family members [61,74,76], whereas situations where siblings have different interests have been described as hindering participation [61]. Overall, the relationship between adolescents with ASD and their siblings seems to be of a similar nature as those in typical sibling relationships, involving overall enjoyment, responsibility, and reciprocity [61]. Hence, siblings remind adolescents with ASD of being similar and different. This is described to play a role in their process of identity formation [61]. Some adolescents with ASD perceive the inequality with their siblings as alienation from their family [61]. The role of a sibling may involve providing a bridging function to the experiences that adolescents with ASD encounter in social roles outside the familial environment. Family members often channel opportunities for social participation [22,55,56] or initiate activities [22,60,62,80]. Participation is hindered when organizational, emotional, or financial limits restrict families in providing these opportunities [60,62,79,80]. An example of this is when a family does not go to the cinema with an adolescent with ASD because family members cannot handle other people’s embarrassment [60]. A higher family income also helps to connect with others [57,65,73,76,78,79,56], as it provides opportunities and flexibility, and makes it easier to offer more options for participation, for example in physical activities [80], or to pay for a case manager [57]. Restricted financial resources might reduce the social participation of families as a whole and consequently that of the adolescents as well. The higher the family income, the greater the frequency of invitations to activities [78]. The educational level of the mother also seems to influence participation [73], as educated mothers might know better how participation can be achieved against the odds. There is a tendency for advocating families to be associated with more positive outcomes [55,67,79,56]. When families fail to facilitate social connectedness or to advocate for adolescents with ASD, this seems to hinder participation [55,59,60,67,72,80].

The second subtheme, “friendships with peers and classmates”, refers to environments provided by classmates at school or peers in the community, which are social partners belonging to similar age groups. For adolescents with ASD, friendships that overcome disability and barriers are the overall goal of participation [56]. If their eagerness fails to elicit responses by peers, participation is hindered [63,81]. A “friend” was described by adolescents with ASD as “someone you can relate to” and “someone you can talk to”, and some of the defining characteristics were trustworthiness, patience, helpfulness, and kindness (p.78) [19]. Friendship is experienced in conjunction with shared interests, shared activities, joy, and enrichment [19,55,74,76,78,56]. Shared interests and shared activities support participation [18,62,74,82]. Adolescents with ASD get to know friends in different situations: at school (51%), in the neighborhood (11%), in sports and leisure activities (9%), from childhood (15%), and through friends of the family (10%) [76]. Over a third of the sample reported spending time with friends engaging in physical activities (37%), watching TV(25%), playing(25%), or conversing (23%). Adolescents with ASD were less likely to do outdoor activities (12%), or “hang out” with friends (8%), engage in artistic activities (4%), or listen to music(4%) [76]. The use of social media also relates to connectedness [79,82]. It prepares for and at the same time reflects social relationships. Twenty-four percent of a sample played video games with peers [82]. From an environmental perspective, it is supporting when peers acknowledge that friendships with adolescents with ASD come about in a different way, and are therefore motivated to adapt [55,66,74,56]. This requires voluntary and reciprocal initiation [55,56]. Respect, often expressed by adolescents with ASD as a wish to be intellectually recognized [74,77,56], seems a good indicator of a supporting environment created by peers or classmates. Participation can be described as successful when friendship with adolescents with ASD is mutually enriching [18,55,74,56]. In these cases, peers and classmates give the adolescents with ASD an insider role and ask about their wishes or preferences. Being allowed to select a partner supports participation in physical education [71]. Developing and systematizing a different communication style supports connectedness [55]. It reveals the importance of humor and allows the adolescents to discover their talents [55,74]. In contrast, even when being included organizationally, adolescents with ASD were described as being isolated or peripheral in classrooms for 71% of the time, and not protected by peers [19,70,81]. This indicates that successful friendship is a support mechanism against bullying [62,67,83].

The third subtheme, “service and staff”, comprises the availability and characteristics of services, and how the staff providing these services influence social connectedness. Overall, receiving a greater number of community services [73,79] or and being educated in a fully or partially inclusive school environment [22] is associated with greater participation in social and recreational activities. But inclusion is not enough, since being equal to other students is important. Thus, authority figures in the school setting must use consistent principles of equity, or equivalence with other students [56]. Administrative flexibility and pedagogic skillfulness influence the way support staff create equity and structure environments to connect adolescents with ASD with their classmates [74,79]. Unprepared, uninformed, and insufficiently skillful support staff hinders [55,63,67,68,77,80]. Such staff members might not be able to deal with challenging behavior, and might even constitute “a negative peer model” [55]. It is not easy to achieve just the right level of support. On the one hand, “over-inclusion”, or a constant level of support, can prevent or block opportunities for interactions with peers [56]. On the other hand, excluding the adolescents, for example due to disruptive relationships with teachers, obviously hinders participation [77]. Overt staff attention can be perceived as negative, as it accentuates the differences [68]. Skilled support staff provide relatedness and immediate reassurance, and serve as role model [55,68,77]. Skilled support should be provided subtly and in the background [55,67,68] to reduce the attention that adolescents with ASD get [74]. Resources to train support staff to work with adolescents with ASD are essential [66,68,72,56]. Lack of collaboration or dysfunctional collaboration between different staff members or between staff and parents has been reported as a hindering factor not only in school settings, but also for optimal care in hospitals [67,68,72]. As regards out-of-school services, only one-third of adolescents with ASD have been reported to participate in inclusive community group activities [78]. A lack of knowledgeable and flexible staff is also reported as a hindering factor when attending physical activities[71,80] or visiting hospitals [72]. Generally, more support and services in the community are required[57]. Others have reported the same for transition to work [66].

The fourth subtheme, “reception of outing and attitudes of environments”, refers to the way disclosure of ASD is handled by the environment, and how attitudes of others influence relationships and thus participation. Many adolescents with ASD express the fear that disclosure of their autism may lead to negative reactions, or even result in stigmatization [60,68,77]. It hinders social participation when a disability is perceived as a weakness and not just as a different form of normality [55]. Adolescents who disclosed their medical diagnosis felt a constant obligation to control other people’s impressions and to show, for example, how “smart” they were [77]. In the context of work, participation is hindered when the presence of ASD implies doing a job less effectively than others [66]. By contrast, sensitively handled disclosure can facilitate understanding, empathy, and positive relationships [60,72,83]. A precondition for positive reception is an attitude that resists labeling and entrenched opinions [55]. Accepting differences implies a negotiation of normalcy. When differences become normal and individualized, which was expressed in one article as “normal for me” (p. 569) [66], this results in a fair chance of equality and support for participation.

### Tension in participation

During the thematic analysis, we discovered ambiguities regarding participation that influenced our attempts to define supportive or hindering environments. In order to still include them in our overview, we distinguished a third main theme, “**tension in participation”**. This refers to dilemmas regarding the participation of adolescents with ASD and is described with three subthemes.

The first subtheme, “isolation and solitary participation”, defined here as being deprived of participation with others, is described by words like “loneliness”, “exclusion” and “bullying” [63,81,83]. On the one hand, adolescents with ASD are socially more isolated [19,61,68,81,83]. Compared to other groups of pupils enrolled in special education, adolescents with ASD are significantly more likely to never see friends (43%), never receive calls from friends (54%), or never be invited to activities (50%) [78]. In another sample[79], 55% had not met up with a friend during the last 12 months. This has also been confirmed by others [57,75]. On the other hand, this should not be confused with the tendency to prefer solitary activities. Frequently reported activities [22] like walking (74%) and engaging in hobbies (41%) can be performed alone. It has also been reported that adolescents with ASD deliberately opt for solitude for reasons of restoration, regeneration, or concentration, which could be regarded as a form of participation for them [22,83].

The second subtheme, “participation in disability-related groups”, refers to friendships and joint activities with other youths with disability. These seem to be frequent among adolescents with ASD. Over half of a sample were reported to have at least one friend with a disability [76] and the probability of belonging to a disability-specific group has been reported as being 25% more likely than among other adolescents [78]. On the one hand, this form of participation provides sameness and identity. Adolescents with ASD provide each other with similar levels of closeness, security, and conflicts, as typically developing classmates [19]. On the other hand, a low community participation rate has been found for adolescents with ASD who participate in disability-related social groups [78]. In any case, there is a tension regarding the function of disability-related groups. They may contribute to the participation of adolescents with ASD, and at the same time hinder their participation in normative groups.

The third subtheme, “normalcy and differences”, refers to the balancing act between “being different” and the need to “fit in”. On the one hand, difference separates, and adolescents with ASD feel a constant negative pressure to conform [68,77]. Ignoring this has negative connotations [68,74,77]. They often try to hide their differences, which in some studies is called “masquerading” [68,77]. This additional effort is described as frustrating and tiresome. Existing norms and enacted attitudes about ways of handling diversity influence the adolescent’s capacity for participation. On the other hand, when “*diversity becomes the norm*” (p.40) [68], pupils have been reported to feel more able to face the challenges and embrace the opportunities of the mainstream school environment [74,56]. Participation is achieved when there is a sense of normalcy, and the diagnosis of ASD ceases to be a person’s main attribute.

### Validation by stakeholders

All seven stakeholders we approached for validation in our study considered their own experiences to be generally reflected by the findings presented to them. Most found the main themes and titles to be a helpful summary of the key environments which impact on adolescents with ASD. However, all commented on aspects which they felt should have been emphasized more, or which they felt were missing. For the sake of clarity, these topics are shown and commented on in Table 5.

**Table 5:** Summary of validation issues mentioned by seven stakeholders.

Who	Topic (E = emphasis, D = disagreement, C = clarification, M = missing)	Addressed in the Review
Three adolescents and young adults with ASD	• Individual differences (C)	• this is true for all results
	• Relationship to siblings is not reciprocal (D)	• “familial environment”?
	• Social skill interventions (M)	• not mentioned
	• Difference between team sports and individual sports (C)	• “social non-participation”, insufficient “staff and services”
	• Bright light and background noise are troublesome (E)	• “physical environment”
	• It is hard to know when somebody is losing interest. (C)	• could be part of “informational environment”
Two parents of adolescents with ASD	• Importance of spiritual communities (E)	• “reception of outing and attitudes”
	• Meaningful employment (M)	• yes, only two studies included
	• More support for all stakeholders (E)	• see discussion
	• More awareness by society is needed (E)	• “reception of outing and attitudes” see discussion on support by parents
	• Support by marriage partner (M)	• we agree
	• The section “Tension of participation” is excellent (E)	• see discussion
	• Protection from negative social environments is insufficiently covered by “security” (C)	• we agree
	• Environments are inter-related (E)	• see discussion
	• “equality but differentness” of ASD (E)	• “normalcy and differences”
	• Phrases like “burden to society”, “disability” and “weaknesses” are disrespectful (D)	• we agreed and removed the first two of these phrases
	• ASD is to be viewed as an ability and not as a disability (C)	• “outing and attitudes”
	• On-going mentorship or “trusted adviser” for work (M)	could be “staff and services”
Two community health professionals: A special needs teacher and an occupational therapist	• Work spaces are often too distracting	• “physical environment”
	• Courses for siblings are needed (E)	• see practical implications
	• Individual sport is not emphasized at school (C)	• could be “services
	• Role of parents needs to be emphasized (E)	• see discussion and practical implications
	• Disclosure is a daily struggle (E)	• “outing and attitudes”
	• Anti-bullying strategies must be visible and noticeable (C)	• “informational environment”
	• Financial resources to train teachers and staff (E)	• could be “staff and services”
	• Apprenticeship and employment are underrepresented (M)	• we agree
	• Relational security seems extremely important (E). If achieved, even body contact is possible (M)	• see discussion, body contact not mentioned
	• Their views are often underrepresented (E)	• see discussion

## Discussion

The aim of this scoping review was to map the existing literature about supporting and hindering environments for the participation of adolescents with ASD. Thus it focuses on the inter-relatedness between environment and participation for this group of young people. To our knowledge, such a synthesis has not been made before.

The number of studies retrieved, published between 2001 and 2018, confirms the large volume of autism-specific research being conducted [2], but the fact that 31% of all scanned abstracts did not meet our age criterion (75% aged between 12–21 years) shows the paucity of research focusing solely on adolescents [84]. This scoping review includes adolescents with ASD with and without co-occurring intellectual disabilities, and represents the full spectrum of support needs, from requiring very substantial support to requiring relatively little support. In line with what was reported by others [7,85], the majority of the abstracts we scanned covered the ICF participation domain of learning and applying knowledge (d1) and the environmental domain of products and technology (e1). Due to our strict inclusion criteria, these domains are poorly represented in the 31 studies, as has been confirmed by Bölte [86]. The 31 included studies mainly addressed three ICF participation domains (d7-d9). The areas of participation among the adolescents with ASD in our study covered mostly real and desired friendships, relationships, leisure activities, and transition. These areas are in line with what was found in other research on autism among adolescents [15,87]. Typical adolescence topics like independence, discovery of public space, sexuality, partnerships, or detachment from parents, were not covered by the research we mapped.

The first main theme we distinguished, “providing security”, is known from disability studies, where quality aspects of participation are described as, *“a sense of security as providing a foundation to pursue challenges and take risks in their lives” (p*. *1450)* [37]. The security function of parents has been widely confirmed [88–90], and it is often reported how the physical environment can hinder the participation of adolescents with ASD [91,92], whereas providing agency and control over the physical environment support it [93,94]. The third subtheme, that of “informational environments” is a novel one. Information affects the security perception of adolescents with ASD, as their ability to derive information from social contexts is reduced [95]. An information-sensitive environment can contribute to their sense of security by providing different and more adapted information. According to the “reasonable person model” [30,31] people can respond more appropriately when their environments support their needs for meaningful, focused, and comprehensive information [39,40]. This allows cognitive mapping, processing of information, and decision making, thus providing a sense of agency and control. This is suitable for adolescents with ASD, who require structure and predictability [96,97]. Used widely in the contexts of urban planning [98,99], this model is also used in fields like fatigue and violence reduction [100]. It might support interventions to improve the participation of adolescents with ASD like orientation and way-finding, building confidence and trust in relationships, or creating restorative places in schools and community settings. Projects regarding architecture and design [101] and community services [102] offer encouraging examples in this respect.

The second main theme, “helping to connect”, mirrors the insider perspective reported in a disability study, in which participation is described as “*a means to experience social connectedness with others and communities*” (p.1459) [37]. Adolescents with ASD often require improvement of their social interaction skills and are perceived as being less interested in social participation [84,103,104]. However, our findings show that adolescents want to be connected and to experience relationships, intimacy, and a sense of belonging. For adolescents with ASD, the “*familial environment*” for participation is just as important as it is for younger children with ASD [105,106]. Compared to typical adolescents, this is not age-appropriate. Wider families need to be supported in their efforts to connect adolescents socially, for example in visiting public spaces, or work internships. This seems a constructive way to meet the needs of caregivers for persons with ASD [107]. Special attention should also be given to siblings, as they can fulfil a bridging function [108]. Courses for siblings of adolescents with ASD can enable them to become allies in social settings, while at the same time they can reflect on and provide feedback on their sibling’s behavior in a way that adolescents are used to and can understand [109]. The reasonable person model can clearly be applied here as well [39,40]. It is the responsibility of the social and attitudinal environment to provide conditions and services to ensure that disclosure of ASD leads to connectedness and acceptance instead of separation.

Acceptance is also addressed in the third main theme, “tension in participation”. The above-mentioned disability study expresses that “*very different patterns of participation can still reflect full participation*” (p.1459) [37]. Although our results address different pattern of participations, including solitary activities [25,110] and participation in disability-related groups, “isolation and solidary participation”is the most striking aspect, as it seems to contradict the term non-participation as used by Hart [36]. Non-participation according to Hart covers the three lowest steps of the participation ladder: “*manipulation*”, “*decoration*”, and “*tokenism*”. Participation, as described here, rarely goes beyond Hart’s “*tokenism*” [36]. Although the adolescents with ASD are apparently given a voice, in fact they have little or no choice in the subject or style of communication, and little or no opportunity to make their own decisions. Results of our study show that it is the role of friend that adolescents with ASD long for. This is appropriate for their age, is strongly expressed, and is similar to what is experienced by other youths with disability [111]. From the perspective of Hart’s theory [36] it might be questioned whether the importance of friendship for adolescents with ASD is sufficiently acknowledged in current service provision and in autism-related research. Similar to what has been found for other disabilities [112], participation in disability-related social groups and friendships between adolescents with ASD allow them to form a positive identity, and to escape the constant pressure to fit in. Recent work suggests that “camouflaging” ASD, or as it is called here “masquerading”, often requires substantial efforts, may lead to stress responses and may have a negative impact on the development of identity [113,114]. This is important for participation, as the normative values that constitute “participation” have not yet been defined or agreed on.

### Limitations and strengths

There are some limitations associated with this review. Due to the overwhelming number of articles we found initially, we did not search any further in the grey literature and performed no new database search in 2018. Relevant data may thereby have been missed. We performed a hand search of the reference lists of the included articles and hand-searched 4 autism-related journals from 2015–2018 to provide current data. There is also the risk of selection bias. Overlaps between the definitions of ICF categories could hardly be solved. Specifically, social relationships (classified in ICF both under participation (d7) and environment (e3)) were impossible to differentiate clearly, as they reciprocally influence each other. As has been proposed by others [28,37], the additional focus on social and occupational roles brought some light into this. Identifying roles makes the demands regarding environments, for example in the role of sibling, more explicit. Our focus on ecological validity led to the exclusion of environmentally based intervention studies. Although most interventions directly or indirectly influence the environment of the adolescent with ASD, studies mostly fail to assess their effect on participation in natural contexts [115] and thus provide no answer to our research question. Methodological strengths lay in the consistent use of the perspectives of adolescents, parents, and peers regarding participation. In addition, the interdisciplinary nature of the research team widened the scope. Furthermore, involving stakeholders to validate the findings and achieve a stakeholder-sensitive, yet scientific presentation is a step rarely reported in scoping studies [49].

### Clinical implications and further research

Practitioners might consider the results of our research for their practice by looking at the environment as a powerful tool to support the participation of adolescents with ASD. As participation is subjective and value-loaded, the adolescents themselves should identify environmental aspects hindering and supporting their participation. The main themes of “security”, “connection” and “tension” can serve as guiding concepts to address possible fields of change. Overall, professionals need to support parents, siblings, peers and staff dealing with adolescents with ASD. Providing relational security enables adolescents with ASD to become risk takers in activities and participation. Professionals need to be careful to prevent further stigmatization. ‘They should address the aspects that make adolescents with ASD different from typically developing adolescents’ without labeling them. Service providers ought rather to focus on staff training, implementation of anti-bullying policy and reviewing the way they provide information to adolescents with ASD.

Further research should focus on acquiring insights into the perceptions and informational requirements of adolescents with ASD regarding participation, in natural contexts, specifically with respect to friendships, out-of-home and out-of-school participation, and work. We further need to research the parents’ strategies and experienced outcomes as regards encouraging participation by influencing environments. The use of the “reasonable person model” for this potentially offers a new way to examine the interaction between participation and the environment in everyday contexts.

## Conclusions

This scoping review shows that there is a complex interrelation between the participation of adolescents with ASD and their environment. Security and connection are the most important environmental aspects regarding the participation of adolescents with ASD and their strong desire for positive peer relationship experiences. Security and connection represent meaningful, subjectively relevant, and feasible aspects of the way in which the environment can shape participation.
